# Post-Marketing Surveillance of Statins—A Descriptive Analysis of Psychiatric Adverse Reactions in EudraVigilance

**DOI:** 10.3390/ph15121536

**Published:** 2022-12-10

**Authors:** Gabriela Pop, Andreea Farcaș, Anca Butucă, Claudiu Morgovan, Anca Maria Arseniu, Manuela Pumnea, Minodora Teodoru, Felicia Gabriela Gligor

**Affiliations:** 1 Preclinical Department, Faculty of Medicine, “Lucian Blaga” University of Sibiu, 550169 Sibiu, Romania; 2 Pharmacovigilance Research Center, “Iuliu Hatieganu” University of Medicine and Pharmacy, 400349 Cluj-Napoca, Romania

**Keywords:** statins, EudraVigilance, psychiatric disorders, psychiatric side effects, safety profile

## Abstract

Statins are included in the category of high-frequency prescription drugs, and their use is on an upward trend worldwide. In 2012, the FDA issued a warning about possible cognitive adverse drug reactions (ADRs) related to statins, some of which are listed in the Summary of Product Characteristics, but there are still concerns about their potential risk of psychiatric events. The aim of this research was to investigate spontaneous reports containing psychiatric ADRs associated with statins by analyzing the EudraVigilance (EV) database. From January 2004 to July 2021, a total of 8965 ADRs were reported for the Systems Organ Class (SOC) “psychiatric disorders”, of which 88.64% were registered for atorvastatin (3659), simvastatin (2326) and rosuvastatin (1962). Out of a total of 7947 individual case safety reports (ICSRs) of the 3 statins mentioned above, in 36.3% (2885) of them, statins were considered the only suspected drug, and in 42% (3338), no other co-administered drugs were mentioned. Moreover, insomnia has been reported in 19.3% (1536) of cases, being the most frequent adverse reaction. A disproportionality analysis of psychiatric ADRs was performed. The Reporting Odds Ratio (ROR) and 95% confidence interval (95% CI) were calculated for simvastatin, atorvastatin and rosuvastatin compared with antiplatelets and antihypertensive drugs. The reporting probability for most ADRs of these statins compared to antiplatelets was higher. The reporting probability for insomnia, nightmares and depression produced by statins compared to antihypertensive drugs was also higher. The results of this analysis augment the existing data about a possible correlation between the administration of statins and the occurrence of psychiatric side effects.

## 1. Introduction

Cholesterol is one of the main factors in the pathogenesis of coronary heart disease and cardiovascular disease (CVD); therefore, the prevention and control of cardiovascular risk by reducing serum LDL cholesterol has become a global therapeutic target.

Statins are included in the category of high-frequency prescription drugs, and their use is on an upward trend worldwide. According to statistics, the prescription of statins over 10 years has increased from 17.9% (2002–2003) to 27.8% (2012–2013). Statins were also found to be used in patients without CVD, such as patients with type 2 diabetes, and those with hyperlipidemia [1,2,3].

Statins are currently the first line of pharmacological therapy for the treatment of hyperlipidemia, and in the primary prevention of coronary and cardiovascular diseases as well as in their secondary prevention. Statins have become one of the most widely prescribed drugs due to their essential role in lowering serum LDL cholesterol, with atorvastatin being ranked the second most commonly administered medications in the United States [4].

The widespread use of statins increases the importance of careful analysis of their adverse effects on the human body. In recent years, despite their good tolerability, concerns have been raised about the neurological side effects of statins [5,6,7].

Although these concerns are based on individual case reports, published studies have presented different, contradictory conclusions [8]. On the one hand, several clinical trials have focused on determining the therapeutic potential of statins in various central nervous system disorders, including dementia, multiple sclerosis (MS), epilepsy, depression and stroke. On the other hand, recent retrospective studies and meta-analysis have explored the development of various neurological disorders secondary to statin treatment [9,10,11].

In 2012, the US Food and Drug Administration (FDA) issued a warning for all statin class of drugs for possible side effects on cognitive performance based on reports from the FDA Adverse Event Reporting System (AERS), as well as literature reviews and randomized post-authorization clinical trials [12]. Furthermore, changes have been made to the Summary of Product Characteristics by including information about the potential cognitive side effects in the adverse reactions section (Table 1).

Although re-assessments of data from clinical trials have identified no evidence of cognitive effects related to statins, further studies have noted that cognitive and psychiatric disorders may sometimes occur [17,18]. There is a variability in the onset of symptoms potentially associated with long-term therapy, which are not severe and reversible, as stated by the FDA. In addition, the FDA has affirmed that these risks do not exceed the cardiovascular benefits of statins [4,12].

The FDA has stressed the need for new information regarding the possible psychiatric effects of all statins. Subsequent studies have focused on tracking neurocognitive side effects induced by statins depending on their solubility profile [19,20,21].

The solubility profile is a key feature that governs the hepatoselectivity of statins and their inhibitory effect on HMG-CoA reductase. Lipophilic statins enter the liver and extrahepatic tissue by passive diffusion through cell membranes, while hydrophilic statins are hepatoselective, and their absorption is mediated by organic anion transporting polypeptide (OATP) transporters. Lovastatin, simvastatin, atorvastatin and fluvastatin are lipophilic statins, while rosuvastatin and pravastatin are categorized as hydrophilic statins. High hepatoselectivity is thought to result in a reduced risk of side effects [22,23].

Paradoxically, statins have also been associated with a reduced risk of dementia and a slowdown in the progression of Alzheimer’s disease. There are studies that have claimed that statins have multiple therapeutic benefits, in addition to their ability to lower serum lipids [24,25,26].

Some researchers believe that statins have immunomodulatory, anti-inflammatory and antioxidant properties, so they can slow down or even prevent changes that lead to disruption of the neuroprogressive cascade and decrease morbidity and mortality related to psychiatric disorders, more so as the leading cause of death in psychiatric disorders remains cardiovascular disease [27].

Based on the abovementioned literature data and regulatory agency communications reporting a risk of cognitive adverse events associated with statins, the aim of this research was to investigate spontaneous reports of psychiatric side effects related to statins by analyzing the EudraVigilance (EV) database, which collects electronic reports of suspected adverse drug reactions for all authorized medicines in the European Economic Area.

## 2. Results

From January 2004 to July 2021, a total of 128,192 reports involving 7 statins (atorvastatin, simvastatin, rosuvastatin, pravastatin, fluvastatin, lovastatin, pitavastatin) were reported in the EV spontaneous reporting system, from 30 countries, both in the European Economic Area (EEA) and Non-European Economic Area (Non-EEA).

The majority of the reports were associated with atorvastatin (N = 59,624, 46.51%; January 2004–July 2021), simvastatin (N = 27,592; 21.52%; December 2004–July 2021) and rosuvastatin (N = 24,846; 19.38%; December 2005–July 2021). For the other statins, the number of reports was considerably lower, respectively, for pravastatin (N = 6812; 5.31%; October 2004–July 2021), fluvastatin (N = 6229; 4.86%; October 2005–July 2021), lovastatin (N = 1844; 1.44%; July 2005–July 2021) and pitavastatin (N = 1245; 0.97%; June 2004–July 2021). This analysis was carried out, for each individual statin, from the date of the first report registered in EV.

**Figure 1:**
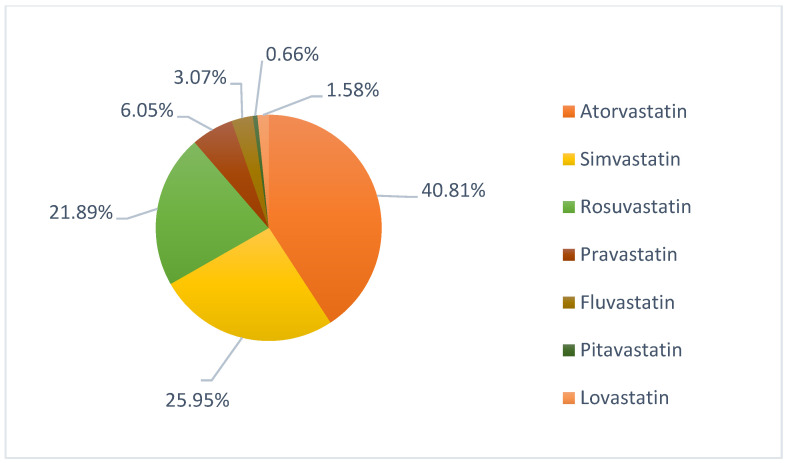
Distribution of suspected psychiatric adverse reactions associated with statins, reported in EudraVigilance.

Of the total reports (N = 128,192) reported in EV for all the statins mentioned, 6.99% (N = 8965) were for the System Organ Class (SOC) “psychiatric disorders.” The majority of these reports were for atorvastatin (N = 3659), simvastatin (N = 2326) and rosuvastatin (N = 1962), accounting for 88.64% of the total reports for this category of adverse reactions, the distribution of which is shown in Figure 1.

Due to the small number of reports associating pravastatin, fluvastatin, lovastatin and pitavastatin with psychiatric adverse reactions, these statins were excluded from further analysis.

According to data presented in Table 2, the majority of reports containing psychiatric adverse reactions were reported by healthcare professionals (N = 5030, 63.29%). The remaining reports were recorded by non-health professionals, with this category including patients, relatives of patients and anyone who was entitled to report the adverse reaction.

Most of the reports associated with reactions in the category of psychiatric disorders referred to patients aged 18–64 years (48.50%) and 65–86 years (35.07%) and were reported in female patients (53.98%). Most adverse reactions were serious (78.14%), while 21.59% were classified as non-serious.

A little more than half of the reports containing psychiatric reactions came from EEA countries (N = 4071, 51.23%), while 48.40% were reported in non-EEA countries.

**Figure 2:**
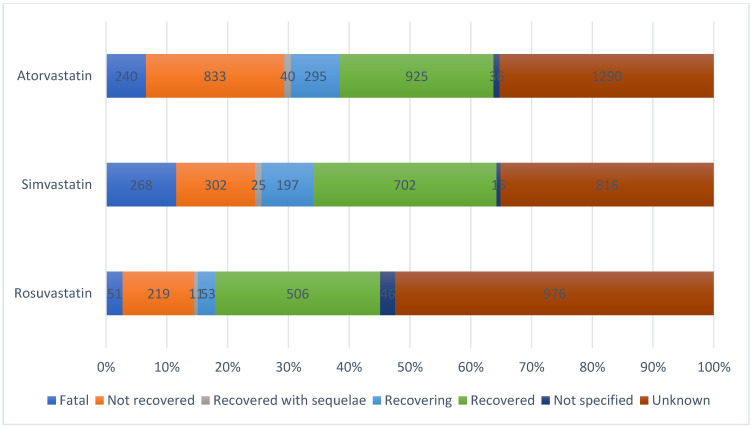
Distribution of reports containing psychiatric side effects associated to atorvastatin, simvastatin and rosuvastatin by outcome.

For most psychiatric ADRs, as it can be seen in Figure 2, the outcome was not reported (40.51%), and for 25.35%, the result was unfavorable (unrecovered/death/recovered with sequelae). A significant percentage of 34.13% were considered reports with a favorable result (recovered/in the process of recovery).

Table 3 shows that out of the 7947 spontaneous reports recorded for atorvastatin, simvastatin and rosuvastatin, in 42.59% (N = 3385) of them, statins were considered the only drugs suspected.

At the same time, for 42% (N = 3338), no other drugs were administered concomitantly with statins.

**Figure 3:**
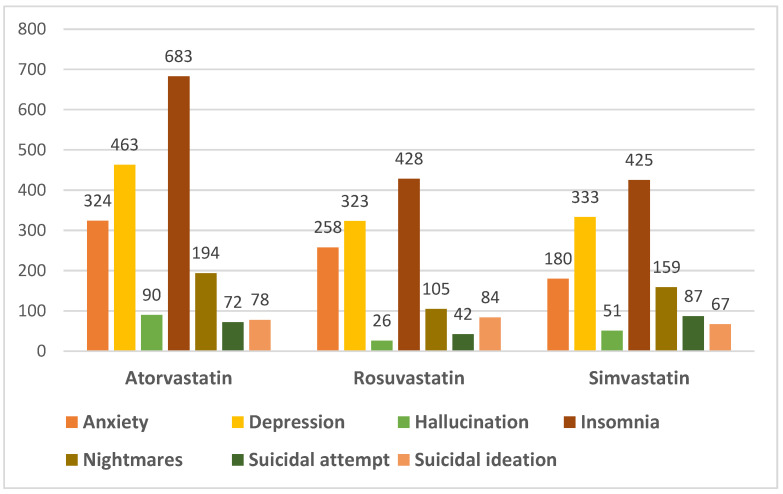
Distribution of the number of reports of psychiatric adverse reactions analyzed for atorvastatin, rosuvastatin and simvastatin.

Out of the seven selected ADRs (anxiety, depression, hallucinations, insomnia, nightmares, suicidal ideation/attempt) that were further evaluated for age, sex and outcome, insomnia (N = 1536, 19.3%) was the most common adverse reaction associated with atorvastatin, simvastatin and rosuvastatin among the adverse reactions observed in this analysis (Figure 3). Insomnia was reported mostly in patients aged 18–64 years (46.94%; N = 721) and 65–85 years (37.70%; N = 579), and especially in females (56.51%; N = 868). The reports that described “insomnia” as an adverse reaction at the psychiatric level showed a favorable result (recovered/in the process of recovery) in 38.15% of cases (N = 586), and for 20.31% (N = 312) of the reports, the adverse reaction was considered “unrecovered” at the time of reporting.

Depression (N = 1119, 14%) was most often reported in patients aged 18–64 years (N = 618; 55.23%), followed by the age group of 65–85 years (N = 322; 28.78%), with most cases being reported in female patients (N = 607; 54.24%). The adverse reaction was associated with a favorable outcome for 36.37% (N = 407) of patients. 

Similarly, anxiety (N = 762, 9.6%) was more commonly reported for the 18–64 age group (N = 424; 55.64%), being an adverse reaction described more in women (N = 487; 63.91%) than men (N = 257; 33.73%). The outcome of this adverse reaction was considered unfavorable at the time of reporting for 19.94% (N = 152) of the cases, while 23.62% (N = 180) of the reports described a favorable result. More than 50% of the reports which referred to “anxiety” associated with statins did not specify the outcome of this condition.

For the abovementioned side effects (insomnia, depression, anxiety), no case was reported in patients under 18 years of age.

Regarding the reporting of nightmares (N = 458, 5.8%) associated with treatment with one of the statins analyzed, the data showed that both patients in the 18–64 age group and those in the 65–85 age category, and both sexes, have reported nightmares following the administration of statins, an adverse reaction with a favorable result in most cases, as it can be seen in Table 4, Table 5 and Table 6.

Hallucinations (N = 167, 2.1%) were reported for all the three statins, especially by those in the age group of 65–85 years (43.71%), both in women (54.49%) and men (40.12%). Case reports were also recorded for the other age groups, among which 28.74% cases were reported for patients aged 18–64 years and, to a lesser extent, 10.78% were reported for those over 85 years.

Suicidal ideation/suicidal attempt are ADRs that have been registered in 430 reports (5.4%) following treatment with atorvastatin, simvastatin or rosuvastatin. In the category of people under 18 years old, 15 cases were mentioned. However, most of the suicidal attempts were in patients in the category of 18–64 years old (56.51%), as well as those in the category of 65–85 years old (20.23%). A total of 22 reports described an unfavorable outcome, including 6 deaths. For most cases (44.19%), this type of adverse reaction had a favorable result.

**Figure 4:**
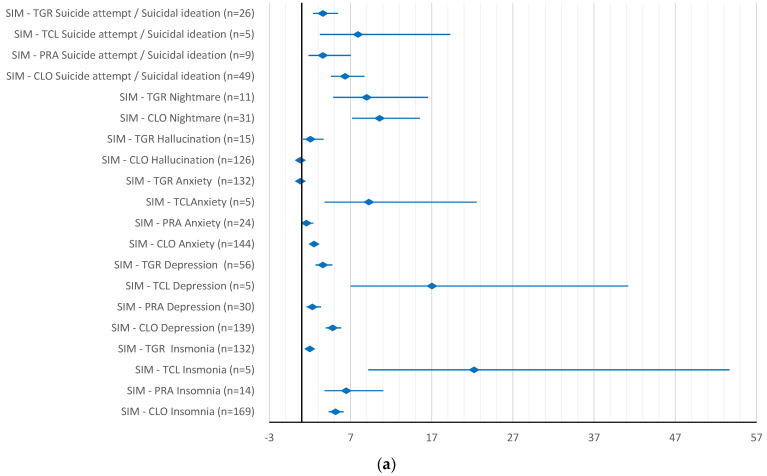

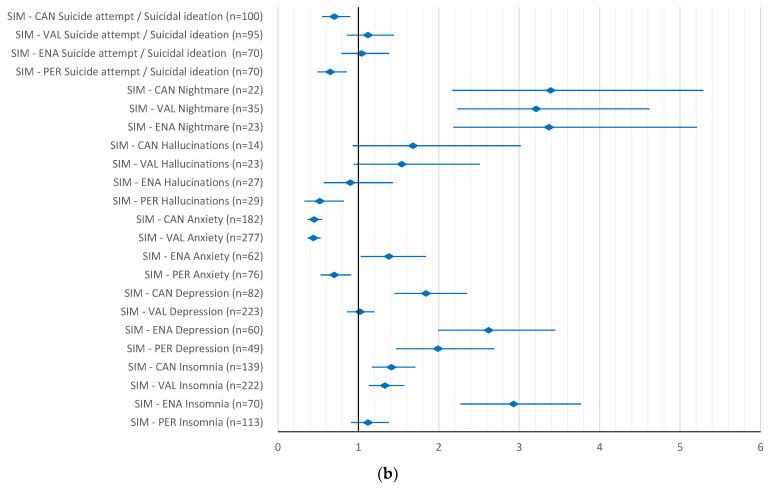
Reporting odds ratio of simvastatin-psychiatric side effects: (**a**) simvastatin—antiplatelet drugs; (**b**) simvastatin—antihypertensive drugs. SIM–simvastatin; CLO–clopidogrel; PRA–prasugrel; TCL–ticlopidine; TGR–ticagrelor; ENA–enalapril; PER–perindopril; CAN–candesartan; VAL–valsartan.

In comparison with antiplatelet drugs, simvastatin had a higher reporting probability for all ADRs evaluated, except for anxiety and hallucinations as compared to ticagrelor (ROR 0.82, 95% CI 0.66–1.03) and clopidogrel, respectively (ROR 0.82, 95% CI 0.59–1.13). However, when compared with antihypertensive drugs, simvastatin had a higher reporting probability for only about half of the ADRs, mainly for insomnia, depression and nightmares (Figure 4a,b).

**Figure 5:**
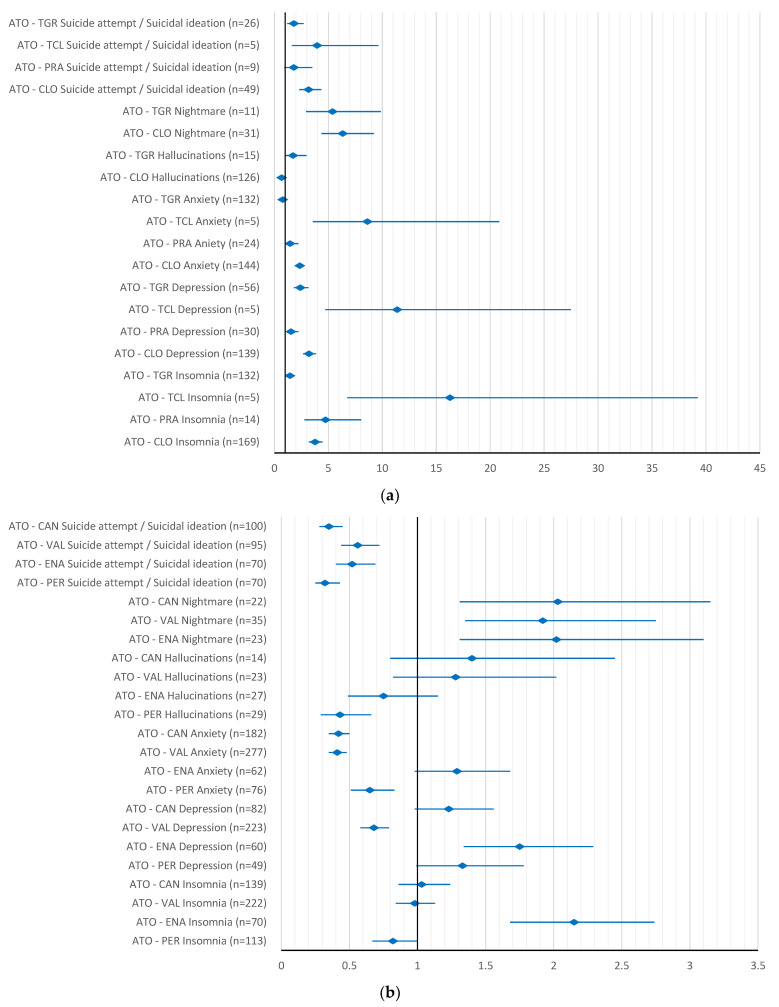
Reporting odds ratio of atorvastatin-psychiatric side effects: (**a**) atorvastatin—antiplatelet drugs; (**b**) atorvastatin—antihypertensive drugs. ATO–atorvastatin; CLO–clopidogrel; PRA–prasugrel; TCL–ticlopidine; TGR–ticagrelor; ENA–enalapril; PER–perindopril; CAN–candesartan; VAL–valsartan.

For atorvastatin, we also found a higher reporting probability for all ADRs when compared to antiplatelet drugs, except for anxiety (ROR 1.46, 95% CI 0.97–2.21) and suicidal attempt/ideation (ROR 1.8, 95% CI 0.92–3.51) when compared to prasugrel, and for hallucinations (ROR 0.68, 95% CI 0.53–0.89) and anxiety (ROR 0.77, 95% CI 0.63–0.94)) related to clopidogrel and ticagrelor, respectively. However, when compared to antihypertensive drugs, a higher reporting probability was found only for nightmares related to enalapril (ROR 2.02,95% CI 1.31–3.1), valsartan (ROR 1.92, 95% CI 1.35–2.75) and candesartan (ROR 2.03, 95% CI 1.31–3.15), and for insomnia (ROR 2.15, 95% CI 1.68–2.74) and depression (ROR 1.75, 95% CI 1.34–2.29) related to enalapril (Figure 5a,b).

**Figure 6:**
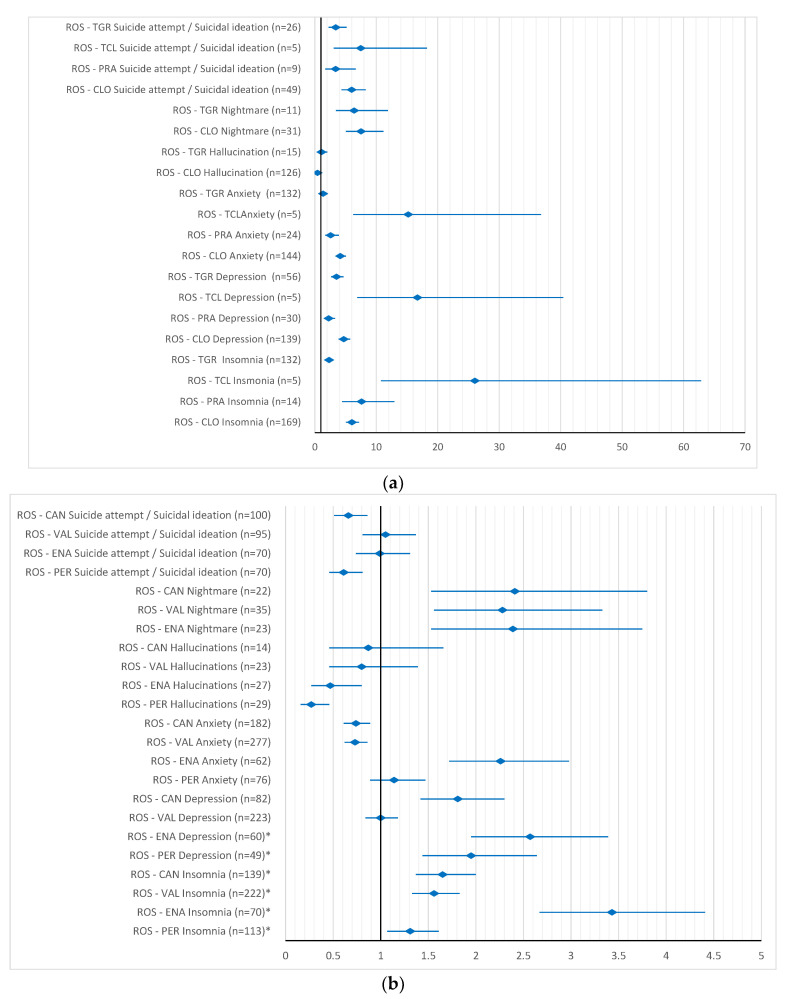
Reporting odds ratio of rosuvastatin—psychiatric side effects: (**a**) rosuvastatin—antiplatelet drugs; (**b**) rosuvastatin—antihypertensive drugs. ROS–rosuvastatin; CLO–clopidogrel; PRA–prasugrel; TCL–ticlopidine; TGR–ticagrelor; ENA–enalapril; PER–perindopril; CAN–candesartan; VAL–valsartan.

Rosuvastatin also had a higher reporting probability for all ADRs when compared to antiplatelet drugs, except for hallucinations related to ticagrelor (ROR 1.08, 95% CI 0.57–2.03) and clopidogrel (ROR 0.43, 95% CI 0.28–0.64). When compared to antihypertensive drugs, for almost half of the ADRs tested, there was a difference in the reporting probability, also mainly for ADRs such as insomnia, depression and nightmare (Figure 6a,b).

## 3. Discussion

Spontaneous adverse drug reporting is used to detect unknown side effects after a drug has been approved, playing an important role in the context of the limitations of clinical trials to detect late and rare adverse reactions. Case reports represent warning signs, which can later trigger interest in in-depth studies for adverse reactions and pharmacovigilance decisions. They mainly serve to generate new hypotheses and signals, but may even provide sufficient evidence to establish a causality between a drug and an adverse event [28].

In this study, we investigated the safety profile of statins regarding psychiatric adverse reactions by analyzing data from the EudraVigilance database. The choice to assess these specific safety issues was determined by post-authorization clinical trials of statins, which reported a reversible effect of cognitive and psychiatric impairment in certain patients [6,7].

According to the literature, lipid-lowering therapies may affect brain function, causing cognitive adverse effects [29]. Statins can affect the brain cholesterol metabolism by lowering the level of plasma cholesterol available. Statins with a lipophilic profile directly influence the metabolism of brain cholesterol, crossing the blood–brain barrier and inhibiting the synthesis of cholesterol in nerve cells. Although the mechanism of transient cognitive dysfunction associated with statins is unknown, Engelberg has proposed a theory that explains the occurrence of adverse psychological and cognitive effects. According to the theory, when cholesterol levels drop in brain cell membranes, this phenomenon leads to lower lipid microviscosity, which may affect neurotransmitter exposure by decreasing synaptic binding and absorption [30].

Because central serotonergic pathways are involved in behavioral control, lower cholesterol levels that occur following treatment with statins or other lipid-lowering drugs may facilitate the occurrence of psychiatric adverse events, including cognitive impairment, acute memory impairment and aggression [7,31,32,33].

At the same time, there are studies that have shown that people with mood disorders may be susceptible to the neuropsychiatric effects of cholesterol-lowering drugs, which justifies further research [34].

The fact that lipid-lowering agents have been found to have similar side effects supports the hypothesis that lowering cholesterol in the brain cell membrane may be an important factor in the etiology of psychiatric reactions. Data from a recent study showed that proprotein convertase subtilisin/kexin type 9 inhibitors (PCSK9Is) have also been associated with a risk of neurocognitive side effects. The results of the study showed that 22.7% of all ICSRs who reported alirocumab or evolocumab as suspicious drugs described the occurrence of neuropsychiatric side effects. However, according to di Mauro et al., a lower reporting probability was found for ICSRs with ADRs belonging to the SOC ‘psychiatric disorders’ for evolocumab and alirocumab versus simvastatin, pravastatin and rosuvastatin [35]. In 2007, the New Zealand Centre for Adverse Reaction Monitoring published an analysis of 364 psychiatric adverse reactions out of a total of 285 reports for statins, fibrates and ezetimibe. Statins have been mentioned most often as the drugs responsible for the occurrence of side effects such as depression, emotional lability, aggression, agitation, nervousness, panic, amnesia, confusion, insomnia and hallucinations. The majority of reports were for simvastatin, with 21% of the total side effects registered, most of which were seen in females. Although the study failed to establish an incidence of psychiatric side effects, it was noted that other lipid-lowering agents have statin-like side effects, and lowering cholesterol in the brain cell membrane may play an important role in the etiology of these types of side effects [36].

Until now, studies that have directly compared lipophilic statins (atorvastatin and simvastatin) and hydrophilic statins (rosuvastatin) in terms of efficacy and safety profile have provided conflicting conclusions. Some researchers have claimed that lipophilic statins with specific pharmacokinetic properties (atorvastatin, simvastatin) are more commonly involved in cognitive adverse events compared to other lipophilic statins and those with hydrophilic properties [37]. Another study that looked at the risk of depression developed after initiating statin treatment found that patients treated with lipophilic statin did not have a statistically significant increase in the risk of developing depression and suicidal ideation compared to those treated with hydrophilic statins. This adverse reaction was found in subgroups of patients with a history of a psychiatric condition [38].

In this study, we found that the number of ICSRs that reported atorvastatin, simvastatin and rosuvastatin as suspected drugs for adverse reactions related to SOC “psychiatric Disorders”, submitted to the EudraVigilance database, was higher than those received for other statins. The increase in ADRs reporting is likely to be related to the frequent use of these three statins. Although the reporting of these adverse reactions has been predominantly performed by healthcare professionals, the high percentage of adverse reactions reported by patients receiving statin treatment is considerable, indicating an awareness of the need to report these symptoms and their impact on patients’ quality of life. Some authors believe that the number of reports issued from patients can be increased by facilitating an independent reporting system and a closer involvement of healthcare professionals in advising the patient on possible adverse reactions and their management [39].

Of the side effects reported in this analysis, insomnia was the most reported for all three statins (N = 1536, 19.3%). Significant signs of sleep disorders associated with statin use were also mentioned in a comprehensive analysis of the FDA Adverse Event Reporting System (FAERS). Statins have been associated with sleep disorders, including sleep onset insomnia, sleep disorders due to a general medical condition and sleep apnea. Among the analyzed statins, simvastatin, atorvastatin and rosuvastatin were the most frequently mentioned [40]. In addition, the purpose of one of the studies published by Tuccori et al. was to test the hypothesis that psychiatric adverse events are associated with the administration of statins by quantitative and qualitative analysis of the signals found in the database of the spontaneous reporting system of adverse reactions in Italy. Of all the psychiatric side effects identified in the analysis, the most frequently mentioned were insomnia, drowsiness, agitation, confusion and hallucinations [41].

According to previous studies [33,38,42], depression was one of the most frequent psychiatric ADRs. The present study identified depression (N = 1119, 14%) as the second most common psychiatric ADR reported in EV for the three statins studied. Of all psychiatric ADRs, the lowest frequency of depression was associated with atorvastatin (12.7%) and the highest frequency with rosuvastatin (16.5%). Otherwise, some researchers have supported the neuroprotective effects of statins [43,44]. The results of a meta-analysis published by Parsaik et al. showed that statin use was associated with a lower risk of depression [45]. Furthermore, a meta-analysis of 7 randomized controlled trials (RCTs) representing 2105 participants displayed no significant differences of overall psychological effects of lovastatin, simvastatin and pravastatin when compared with placebo. When the outcomes on depression and mood were analyzed separately, significant improvements of statins on mood scores were observed, highlighting a potential benefit of statins in mood-related disorders due to their antioxidative and anti-inflammatory effects [46]. In addition, another meta-analysis of 10 RCTs representing 2517 participants displayed significant improvements on depression scores in patients receiving simvastatin, lovastatin, atorvastatin or pravastatin, compared with placebo. A subsequent subgroup analysis revealed the beneficial effect of statin use in reducing depression symptoms in patients with clinical depression, while in patients without depression, the difference was not significant [47].

The ICRSs included anxiety as another ADR associated with atorvastatin, simvastatin and rosuvastatin treatment (N = 762). A higher frequency of anxiety was in the female group (63.91%). Over 20% of cases were not recovered at the time of reporting. 

Suicidal ideation and suicidal attempt (N = 430) represent 5.41% of all psychiatric ADRs reported for atorvastatin, simvastatin and rosuvastatin. Moreover, according to the ICRSs, suicide occurred in six cases.

Another qualifying descriptor for the present analysis was age. Thus, we found that psychiatric side effects affected both age categories of 18–64 years (atorvastatin–47.99%, simvastatin–49.66% and rosuvastatin–48.06%) and 65–85 years (atorvastatin–35.09%, simvastatin–34.82% and rosuvastatin–35.32%). This can be expected as both the elderly and adult populations can be affected by hypercholesterolemia, with recommendations for statin therapy in accordance with the existing guidelines [48]. Hynuah Kim et al. analyzed national pharmacovigilance data associated with statin use in Korea (KAERS), and for the elderly (>65 years), dizziness was the second most common adverse reaction reported after myalgia, and insomnia and asthenia were reported more frequently in the 18–64 age group [49].

A slightly higher frequency of psychiatric ADRs in females (53.98%) compared to males was observed in the present study. According to the literature, hypercholesterolemia appears to be more common in women, especially after menopause, a period that has been shown to be associated with an increase in total cholesterol and LDL cholesterol [50,51,52]. A prospective cohort study also found that females more often describe symptoms of anxiety than men, a factor that influences adherence to treatments, including statin therapy that may be compromised [53]. Furthermore, behavioral and psychiatric changes such as nightmares and aggression, even the idea of suicide associated with statin treatment, were analyzed in a randomized study, which aimed to investigate whether there was a correlation between these side effects and low testosterone levels [54]. It has been mentioned in the literature that statins can lower testosterone in both men and women [55]. In the abovementioned study, it was observed that women showed increased aggression, especially those over 45 years old [54]. These results are consistent with the results of the WISE study, which looked at the behavior of women treated with cholesterol-lowering drugs [56].

Regarding the seriousness of ADRs, 78.14% of the reported ADRs were classified as serious. Neuropsychiatric ADRs may include various signs and symptoms, ranging from mild ADRs, including sleep disturbances, abnormal dreams, dizziness, loss of balance and tinnitus, to more severe ADRs such as depression, suicide, seizures and paralysis [57].

All three statins showed a higher reporting probability for most ADRs tested when compared to antiplatelet drugs. However, when compared to antihypertensive drugs, the reporting probability was lower for about half of the reactions tested, except for sleep disturbances such as insomnia and nightmares, and depression. Although the use of antiplatelet or antihypertensive drugs in patients with hypercholesterolemia can be common due to co-morbidities, in almost half of the ICSRs evaluated in the present study, there were no concomitant drugs and/or there was no other suspected or interacting drug. Still, for the other half of ICSRs, the role of concomitant medications in the occurrence of these psychiatric ADRs cannot be excluded.

### Limitations of This Study

The spontaneous reporting system is affected by limitations that are mainly related to under-reporting and the inaccuracy of information. Some important information was not available in the aggregated data evaluated from adrreports.eu (comorbidities, previous/current medical conditions, concomitant medications, duration of therapy, etc.). All unreported information could have affected the correct classification of the adverse reactions described.

Furthermore, a comparison between statins could not be made, as reports in which several statins mentioned as suspected drugs were not excluded from the analysis and data from spontaneous reporting system were not appropriate for such direct comparisons.

Moreover, the number of case reports for a particular drug or suspected adverse reaction depends not only on the actual frequency of the adverse reaction, but also on the extent and conditions of use of the drug, the nature of the reaction and the awareness of patients. Therefore, comparing the number of case reports between drugs can provide a distorted picture of their safety profiles.

ROR could not be used to quantitatively determine the risk of ADRs as it is a simple indicator of potential safety issues. In addition, the total number of patients using statins is not known, so the incidence and prevalence of their ADRs could not be calculated.

The data publicly available and analyzed from adrreports.eu do not allow the scientific evaluation of the cause–effect relationship between the target drug and the suspected adverse reaction. The nature of causation is part of a broad benefit–risk monitoring process, so information about the patient’s medical condition and medical history is needed to establish causation.

## 4. Materials and Methods

### 4.1. Study Design

The study was conducted based on the analysis of electronic reports containing suspected adverse reactions associated with statins, submitted to EudraVigilance (EV), from January 2004 to 3rd of July 2021.

Data were extracted from the adrreports.eu portal, the European Database of suspected adverse drug reaction reports [58].

Spontaneous adverse reactions were reported in the EV database by both EEA and non-EEA regulators, marketing authorization holders, health professionals or patients [59].

The regulations for data protection were not necessary, and the present study did not involve the approval of the ethics board because the analysis included non-identifiable persons. Moreover, the data extracted from ICSRs did not contain personal information [60].

### 4.2. Material

All ICSRs containing at least one adverse reaction in the category “psychiatric disorders” of the System Organ Class (SOC), according to the terminology of the Medical Dictionary for Regulatory Activities (MedDRA), in association with at least one of the following statins: atorvastatin, simvastatin, rosuvastatin, pravastatin, fluvastatin, lovastatin or pitavastatin, were extracted from EV. Then, the analysis focused on the following suspected reactions from the category “psychiatric disorders:” anxiety, depression, hallucinations, insomnia, nightmares, suicidal ideation and suicide attempt reported in association with atorvastatin, simvastatin and rosuvastatin.

### 4.3. Data Analysis

Out of the information extracted, we analyzed patients’ age, sex, geographical origin, number of cases recorded over time, number of individual cases by reaction group, as well as data on reporters and outcome and seriousness.

An adverse reaction was classified as serious if (1) it resulted in death, (2) it was life-threatening, (3) it required hospitalization or prolongation of existing hospitalization, (4) it resulted in persistent or significant disability/incapacity, (5) it was a congenital anomaly/birth defect, or (6) it had the consequence of manifesting other important medical conditions [61].

All reports were included in the database, regardless of causality or seriousness.

Subsequently, the cases describing psychiatric adverse reactions associated with the use of statin were analyzed for the following adverse reactions (preferred terms PT’s): anxiety, depression, hallucinations, insomnia, nightmares and suicidal ideation/attempt. Three web reports were available for these reactions, the first report showing the data by age group and sex, the second by the group of reporters and the third by the outcome of the adverse reaction.

For the disproportionality analysis, we calculated the Reporting Odds Ratio (ROR) and 95% confidence interval (95% CI) based on a classification of the individual cases in the database into four categories and two dichotomous variables (a two-by-two contingency table) as recommended to be used in the EV system [62]. The ROR was estimated for the psychiatric ADRs (PT’s) such as anxiety, depression, hallucinations, insomnia, nightmares and suicidal ideation/attempt. For the analysis of the individual 3 statins (simvastatin, atorvastatin and rosuvastatin), we used antiplatelet (clopidogrel, prasugrel, ticlopidine and ticagrelor) and antihypertensive (enalapril, perindopril, valsartan and candesartan) drugs as reference, restricting the comparator background to drugs used in common therapeutic areas and in similar clinical contexts [63]. A signal of disproportionate reporting was assumed when a case count of ≥5 in EV and a lower 95% confidence interval of the ROR of >1.0 was found, as recommended within the EU [62]. For all analyses, we included all reports with the abovementioned ADRs and drugs, regardless of the drug role as suspect, interacting or concomitant. Data extraction for the disproportionality analysis was performed on 14 November 2022.

## 5. Conclusions

Psychiatric side effects such as depression, anxiety, insomnia, nightmares, hallucinations, suicidal ideation and suicide attempts, that were spontaneously reported in association with statin use, were analyzed in this study, of which insomnia was the most common. For all statins, the 18–64 age group was most often involved. Most cases have been reported by healthcare professionals; therefore, information from spontaneous reports of suspected adverse reactions can be taken into account and interpreted in a scientific context for the future in-depth analysis of this data. Educating patients about these potential risks and maintaining vigilance in clinical investigations of unusual side effects are key factors in increasing the safety of statins. Careful monitoring of possible statin-induced psychiatric symptoms after long-term administration should be part of any ongoing assessment of evidence obtained from clinical case reports.

## Figures and Tables

**Table 1:** Psychiatric disorders included in the summary of product characteristics.

Name	Active Substance	Undesirable Effects	Frequencies *	Reference
CRESTOR^®^ (5 mg, 10 mg, 20 mg, 40 mg)	Rosuvastatin	Depression	Not known	[13]
SORTIS^®^ (10 mg, 20 mg, 40 mg, 80 mg)	Atorvastatin	Nightmares	Uncommon	[14]
		Insomnia	Not known	
		Depression	Not known	
ZOCORD^®^ (10 mg, 20 mg, 40 mg)	Simvastatin	Insomnia	Very rare	[15]
		Depression	Not known	
		Sleep disturbance	Not known	
		Nightmares	Not known	
LESCOL^®^ (20 mg, 40 mg, 80 mg)	Fluvastatin	Insomnia	Common	[16]
		Memory loss	Not known	
		Sleep disturbance	Not known	
		Nightmares	Not known	
		Depression	Not known	

* The frequencies of adverse events are ranked according to the following: Common (>1/100, <1/10); Uncommon (>1/1000, <1/100); Rare (>1/10,000, <1/1000); Very rare (<1/10,000); Not known (cannot be estimated from the available data).

**Table 2:** Characteristics of reports of spontaneous statin reactions, included in the SOC “psychiatric disorders”, recorded in EudraVigilance (January 2004–July 2021).

		Atorvastatin n = 3659 N, (%)	Simvastatin n = 2326 N, (%)	Rosuvastatin n = 1962 N, (%)
Age category	18–64 years	1756 (47.99)	1155 (49.66)	943 (48.06)
	65–85 years	1284 (35.09)	810 (34.82)	693 (35.32)
Gender	Male	1522 (41.60)	1111 (47.76)	805 (41.03)
	Female	2039 (55.73)	1129 (48.54)	1122 (57.19)
Reporter group	Health professionals	2172 (59.36)	1732 (74.46)	1126 (57.39)
	Non-health professionals	1422 (38.86)	573 (24.63)	835 (42.56)
Seriousness	Serious ADRs	2911 (79.56)	1718 (73.86)	1581 (80.58)
	Non-serious ADRs	740 (20.22)	596 (25.62)	380 (19.37)
Countries	EEA	2175 (59.44)	1294 (55.63)	602 (30.68)
	Non-EEA	1454 (39.74)	1032 (44.37)	1360 (69.32)

**Table 3:** Number of ICSRs without other suspected/interacting/concomitant drugs.

Statins	Number of ICSRs That Do Not Mention Another Suspected Drug/Interacting Drug (%)	Number of ICSRs That Do Not Mention Another Concomitant Drug (%)
Atorvastatin	1983 (54.20)	1765 (48.24)
Simvastatin	557 (23.95)	809 (34.78)
Rosuvastatin	845 (43.07)	764 (38.94)

**Table 4:** Psychiatric side effects for atorvastatin.

**Atorvastatin**						
Type of side effects	Anxiety n = 324 N, (%)	Depression n = 463 N, (%)	Hallucination n = 90 N, (%)	Insomnia n = 683 N, (%)	Suicidal attempt/ideation n = 150 N,(%)	Nightmares n = 194 N, (%)
**Age Group N (%)**						
Not specified	30 (9.26)	62 (13.39)	15 (16.67)	88 (12.88)	21 (14.00)	24 (12.37)
<18 years	0	0	0	0	7 (4.67)	0
18–64 years	196 (60.49)	248 (53.56)	22 (24.44)	309 (45.24)	87 (58.00)	77 (39.69)
65–85 years	94 (29.01)	139 (30.02)	40 (44.44)	264 (38.65)	32 (21.33)	87 (44.85)
>85 years	2 (0.62)	14 (3.02)	13 (14.44)	22 (3.22)	2 (1.33)	6 (3.09)
**Sex N (%)**						
M	102 (31.48)	210 (45.36)	37 (41.11)	286 (41.87)	68 (45.33)	93 (47.94)
F	218 (67.28)	243 (52.48)	48 (53.33)	376 (55.05)	77 (51.33)	99 (51.03)
Not specified	4 (1.23)	10 (2.16)	5 (5.56)	21 (3.07)	5 (3.33)	2 (1.03)
**Outcome N (%)**						
Not recovered	79 (24.38)	80 (17.28)	9 (10.00)	147 (21.52)	1 (0.67)	41 (21.13)
Recovered	47 (14.51)	115 (24.84)	32 (35.56)	196 (28.70)	70 (46.67)	79 (40.72)
Recovered with sequelae	11 (3.40)	6 (1.30)	2 (2.22)	8 (1.17)	0	2 (1.03)
Recovering	19 (5.86)	48 (10.37)	5 (5.56)	70 (10.25)	11 (7.33)	13 (6.70)
Fatal	0	2 (0.43)	0	0	2 (1.33)	0

**Table 5:** Psychiatric side effects for simvastatin.

**Simvastatin**						
Type of side effect	Anxiety n = 180 N, (%)	Depression n = 333 N, (%)	Hallucination n = 51 N, (%)	Insomnia n = 425 N, (%)	Suicidal attempt/ideation n = 154 N, (%)	Nightmares n = 159 N, (%)
**Age Group N (%)**						
Not specified	23 (12.78)	50 (15.02)	7 (13.73)	60 (14.12)	27 (17.53)	16 (10.06)
<18 years	0	0	1 (1.96)	0	3 (1.95)	0
18–64	94 (52.22)	183 (54.95)	20 (39.22)	215 (50.59)	91 (59.09)	64 (40.25)
65–85	58 (32.22)	94 (28.23)	21 (41.18)	148 (34.82)	30 (19.48)	72 (45.28)
>85	5 (2.78)	6 (1.80)	2 (3.92)	2 (0.47)	3 (1.95)	0
**Sex N (%)**						
M	58 (32.22)	162 (48.65)	24 (47.06)	178 (41.88)	72 (46.75)	82 (51.57)
F	110 (61.11)	154 (46.25)	25 (49.02)	237 (55.76)	74 (48.05)	75 (47.17)
Not specified	12 (6.67)	5 (1.50)	2 (3.92)	10 (2.35)	8 (5.19)	2 (1.26)
**Outcome N (%)**						
Not recovered	23 (12.78)	56 (16.82)	4 (7.84)	73 (17.18)	6 (3.90)	0
Recovered	40 (22.22)	103 (30.93)	22 (43.14)	132 (31.06)	48 (31.17)	2 (1.26)
Recovered with sequelae	4 (2.22)	4 (1.20)	0	6 (1.41)	3 (1.95)	4 (2.52)
Recovering	20 (11.11)	42 (12.61)	1 (1.96)	44 (10.35)	14 (9.09)	13 (8.18)
Fatal	0	0	0	0	4 (2.60)	0

**Table 6:** Psychiatric side effects for rosuvastatin.

**Rosuvastatin**						
Type of side effect	Anxiety n = 258 N, (%)	Depression n = 323 N, (%)	Hallucination n = 26 N, (%)	Insomnia n = 428 N, (%)	Suicidal attempt/ideation n = 126 N, (%)	Nightmares n = 105 N, (%)
**Age Group N (%)**						
Not specified	11 (4.26)	30 (9.29)	5 (19.23)	46 (10.75)	31 (24.60)	11 (10.48)
<18 years	0	0	0	0	5 (3.97)	0
18–64	134(51.94)	187 (57.89)	6 (23.08)	197(46.03)	65 (51.59)	39 (37.14)
65–85	102(39.53)	89 (27.55)	12 (46.15)	167(39.012	25 (19.84)	52 (49.52)
>85	11 (4.26)	16 (4.95)	3 (11.54)	18 (4.21)	0	3 (2.86)
**Sex N (%)**						
M	97 (37.60)	105 (32.51)	6 (23.08)	163(38.08)	57 (45.24)	50 (47.62)
F	159(61.63)	210 (65.02)	18 (69.23)	255(59.58)	66 (52.38)	54 (51.43)
Not specified	2 (0.78)	8 (2.48)	2 (7.69)	10 (2.34)	3 (2.38)	1 (0.95)
**Outcome N (%)**						
Not recovered	35 (13.57)	44 (13.62)	5 (19.23)	92 (21.50)	6 (4.76)	19 (18.10)
Recovered	51 (19.77)	68 (21.05)	7 (26.92)	109 (25.47)	42 (33.33)	48 (45.71)
Recovered with sequelae	0	2 (0.62)	0	3 (0.70)	0	1 (0.95)
Recovering	18 (6.98)	31 (9.60)	1 (3.85)	35 (8.18)	5 (3.97)	10 (9.52)
Fatal	0	0	0	0	0	0

## Data Availability

Publicly available datasets were analyzed in this study. This data can be found here: https://www.adrreports.eu/ (accessed on 3 July 2021 and 14 November 2022).
